# Effects and Mechanism of Atmospheric-Pressure Dielectric Barrier Discharge Cold Plasma on Lactate Dehydrogenase (LDH) Enzyme

**DOI:** 10.1038/srep10031

**Published:** 2015-05-20

**Authors:** Hao Zhang, Zimu Xu, Jie Shen, Xu Li, Lili Ding, Jie Ma, Yan Lan, Weidong Xia, Cheng Cheng, Qiang Sun, Zelong Zhang, Paul K. Chu

**Affiliations:** 1 School of Life Science, University of Science and Technology of China, Hefei, Anhui Province 230026, People's Republic of China; 2 Department of Thermal Science and Energy Engineering, University of Science and Technology of China, Hefei, Anhui Province 230026, People's Republic of China; 3 Institute of Plasma Physics, Chinese Academy of Sciences, P. O. Box 1126, Hefei 230031, P. R. China; 4 Center of Medical Physics and Technology, Hefei Institutes of Physical Science, Chinese Academy of Sciences, Hefei 230031, People's Republic of China; 5 Department of Physics and Materials Science, City University of Hong Kong, Tat Chee Avenue, Kowloon, Hong Kong, China

## Abstract

Proteins are carriers of biological functions and the effects of atmospheric-pressure non-thermal plasmas on proteins are important to applications such as sterilization and plasma-induced apoptosis of cancer cells. Herein, we report our detailed investigation of the effects of helium-oxygen non-thermal dielectric barrier discharge (DBD) plasmas on the inactivation of lactate dehydrogenase (LDH) enzyme solutions. Circular dichroism (CD) and dynamic light scattering (DLS) indicate that the loss of activity stems from plasma-induced modification of the secondary molecular structure as well as polymerization of the peptide chains. Raising the treatment intensity leads to a reduced alpha-helix content, increase in the percentage of the beta-sheet regions and random sequence, as well as gradually decreasing LDH activity. However, the structure of the LDH plasma-treated for 300 seconds exhibits a recovery trend after storage for 24 h and its activity also increases slightly. By comparing direct and indirect plasma treatments, plasma-induced LDH inactivation can be attributed to reactive species (RS) in the plasma, especially ones with a long lifetime including hydrogen peroxide, ozone, and nitrate ion which play the major role in the alteration of the macromolecular structure and molecular diameter in lieu of heat, UV radiation, and charged particles.

Owing to advantages such as production of highly reactive species at low temperature and flexible operation^1^,^2^, atmospheric-pressure non-thermal plasmas have attracted much attention in biology and biomedicine^3^ and applications include plasma sterilization^4^,^5^,^6^,^7^,^8^,^9^,^10^,^11^, living tissue treatment^12^, blood coagulation^13^, cell detachment^14^,^15^, induction of apoptosis^16^,^17^,^18^, cell proliferation^19^, cancer therapy^20^,^21^,^22^,^23^,^24^, and so on. However, even though the biological effects of atmospheric-pressure plasmas have been investigated and several possible mechanisms have been suggested, systematic verification of these hypotheses is still lacking and the precise mechanism is still not well understood. To gain further insight, it is necessary to study not only the biological effects of cells and tissues, but also their interaction with cold plasmas on the molecular level. Proteins are the main vehicles of biological functions and account for 68% of the dry weight of cells and tissues. There have been investigations on the use of atmospheric-pressure plasmas to modify the secondary structure of proteins in aqueous solutions and inactivate infectious prion proteins under dry conditions. For example, discharge plasmas inactivate and induce Heme degradation of horseradish peroxidase in the phosphate buffer (PBS) solution^25^, inactivates lysozyme in an aqueous solution^26^, activates lipase in the PBS solution^27^, and inactivates polyphenoloxidase (PPO) and peroxidase (POD) in a model food system^28^. However, in spite of recent progress, the molecular mechanism between plasmas and enzymatic activity is still unclear. In this work described in this paper, a dielectric barrier discharge (DBD) plasma is used to treat lactate dehydrogenase (LDH), an important sugar metabolic enzyme^29^,^30^ and the mechanism of protein inactivation and effects on cell metabolism are investigated.

## Results and discussion

### Emission spectrometry and mass spectrometry of DBD plasmas

**Figure 1:**
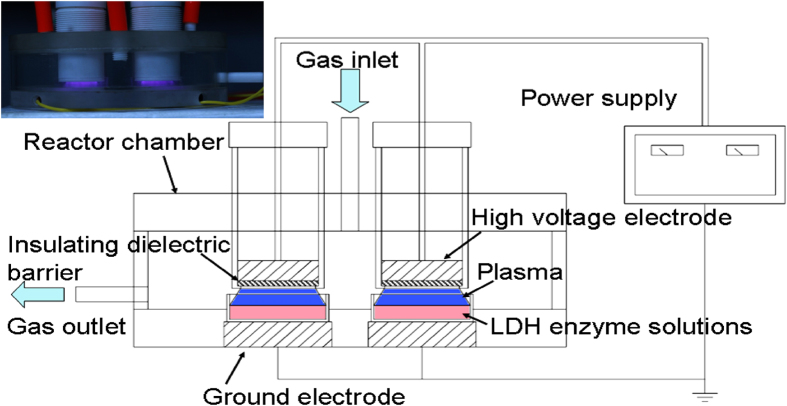
The atmospheric-pressure DBD plasma device.

**Figure 2:**
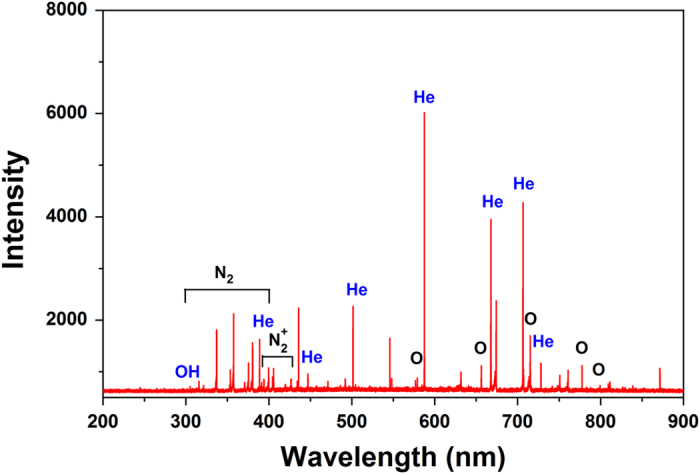
Optical emission spectra of the helium-oxygen DBD plasma.

The typical optical spectrum of the helium-oxygen DBD plasma (Fig. 1) between 200 and 900 nm is displayed in Fig. 2. The dominant emission lines illustrate the presence of the metastable helium atom He (728.1 nm, 706.5 nm, 667.8 nm, 587.5 nm, 501.5 nm, 447.1 nm and 388.8 nm), OH radical (306-310 nm), and atomic oxygen (OI: 799.5 nm, 777.2 nm, 715.7 nm; OII: 656.5 nm, and 578.4 nm). In addition, the detected reactive species associated with nitrogen are excited nitrogen molecules between 300 and 400 nm.

**Figure 3:**
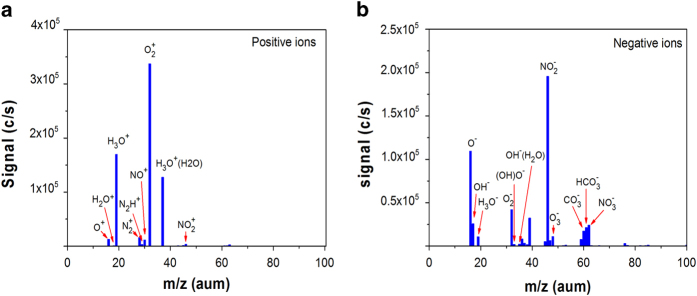
Mass spectra of the helium-oxygen DBD plasma: (**a**) Positive ions, (**b**) Negative ions.

Figures 3(a) depict the time-averaged mass spectra of positive and negative ions obtained at a distance of 5 mm from the bottom of the quartz glass DBD plasma to the orifice of the mass spectrometer. Ions up to 100 amu are detected. The positive mass spectrum in Fig. 3a shows about there are 10 predominant species in the helium-oxygen plasma, namely O_2_^+^, H_3_O^+^, H_3_O^+^(H_2_O), N_2_^+^, O^+^, NO^+^, as well as small portions of H_2_O^+^, N_2_H^+^, NO_2_^+^. In the negative spectrum in Fig. 3(b), more than 10 species are detected and the main species are O^−^, OH^−^, H_3_O^−^, O_2_^−^, (OH)O^−^, OH^−^(H_2_O), NO_2_^−^, O_3_^−^, and NO_3_^−^.

The DBD plasma produces a variety of ions and free radicals in the gas phase. These active ions and free radicals react with water and produce various biologically active reactive species (RS) in the liquid phase such as ones with a long lifetime including hydrogen peroxide (H_2_O_2_), ozone (O_3_), and nitrate ion (NO_3_^−^) as well as short-lived RS including hydroxyl radical (OH^−^), superoxide (O_2_^−^), and singlet oxygen^31^. Ozone is produced in the DBD plasma by the interaction between atomic oxygen and oxygen in the gas phase and then spreads into the liquid phase. Oxygen reduction by two electrons forms hydrogen peroxide, while the nitrate ion is produced by the reaction between nitrogen dioxide and water. In this study, some known long-lived species in the liquid are measured and the possible production mechanism is shown in Table 1. RS is highly reactive which can mediate the bio-macromolecules *via* the cell membrane or intercellularly. By comparing the direct treatment of LDH with indirect treatment, the effects of the short-lived RS in the liquid phase can be explored.

### Concentration of RS

**Figure 4:**
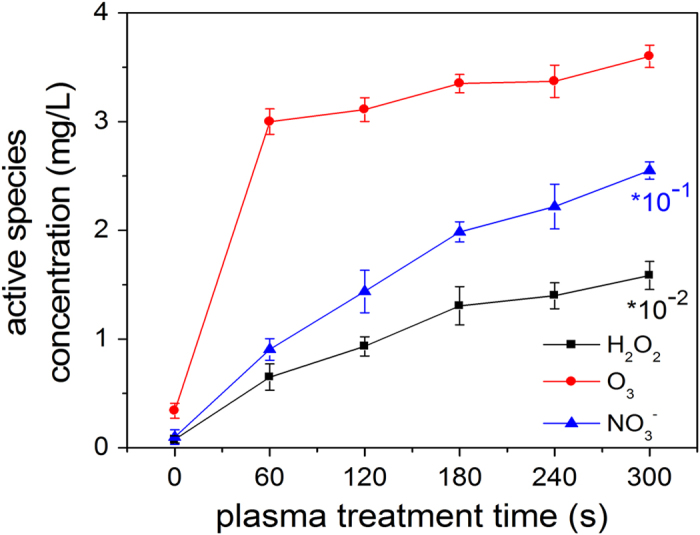
Concentrations of reactive species as a function of treatment time between 0 and 300 s.

The sample solutions were treated by the non-thermal DBD plasma. Since the LDH enzyme is dissolved in PBS, the RS in PBS created by the plasma are expected to affect the LDH enzyme. Figure 4 shows the RS concentrations including those of ozone, hydrogen peroxide, and nitrate ion for different treatment time. The RS concentrations increase with the plasma exposure time. The hydrogen peroxide, nitrate ion, and ozone concentrations increase from 0.077 mg/L to 158.5 mg/L, 0.098 mg/L to 25.5 mg/L, and 0.34 mg/L to 3.6 mg/L, respectively, after 300 s.

**Figure 5:**
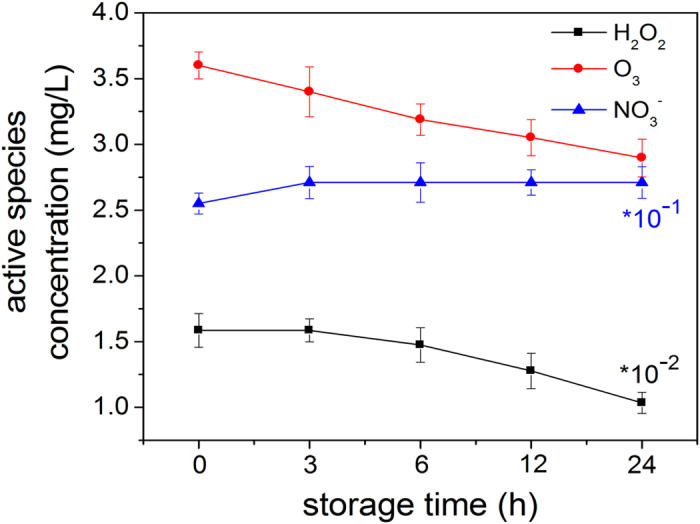
Concentrations of reactive species after plasma treatment for 300 s treatment and different storage time from 0 to 24 h.

The RS concentrations after storage (0 to 24 h) are monitored as shown in Fig. 5 which shows two different trends for the three kinds of long-lived RS. For ozone and hydrogen peroxide, the concentrations decrease within 24 h. The concentration of ozone drops from 3.6 mg/L to 2.9 mg/L whereas that of hydrogen peroxide diminishes from 158 mg/L to 103.5 mg/L. However, the nitrate ion content rises from 25.5 mg/L to 27.1 mg/L in the first 3 hours possibly due to oxidation of nitrite ions and then stabilizes in the remaining time.

### Inactivation of LDH by plasma treatment

**Figure 6:**
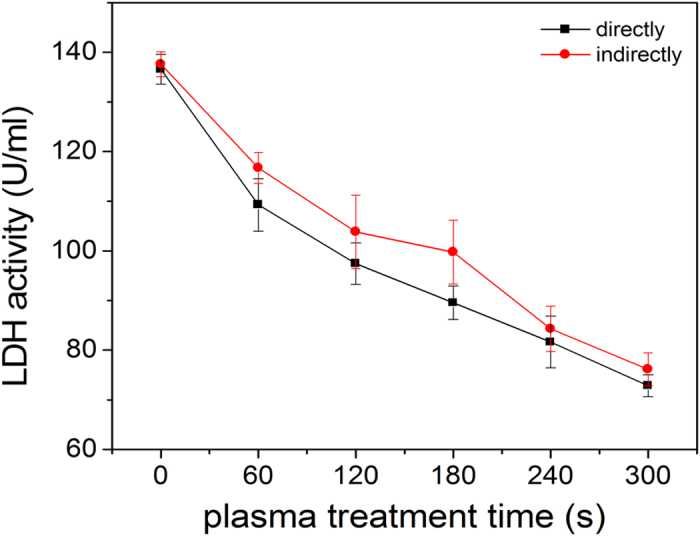
Plasma inactivation kinetics of LDH in the direct and indirect modes for different treatment time from 0 to 300 s.

The LDH protease solution is exposed to the helium-oxygen non-thermal dielectric barrier discharge plasma directly and indirectly and the absolute LDH activity is assessed after treatment for one hour. As shown in Fig. 6, the LDH activity decreases steadily with exposure time regardless of treatment modes revealing that inactivation of the LDH activity increases with time. Compared to the direct treatment, reduction of the LDH activity is less in the indirect treatment for the same time. However, as reported previously on lipase treated by a plasma jet^27^,^32^, there is no difference in the enzymatic activity between the direct and indirect treatment. This may be due to the special physical and chemical properties of proteins and difference in the sensitivity of different proteins for different plasma. In our study, the difference in LDH enzymatic activity between the direct treatment and indirect treatment can be attributed to the difference in the RS in the two modes. Compared to the indirect treatment, besides the effects of the long-lived RS (hydrogen peroxide, ozone, and nitrate ions in the PBS solution), there are short-lived RS such as OH˙, O_2_^−^, and O_2_ (^1^Δg) as well as UV in the direct treatment^31^,^33^,^34^,^35^,^36^. Hence, the RS may be responsible for the slightly larger effects in the direct treatment.

**Figure 7:**
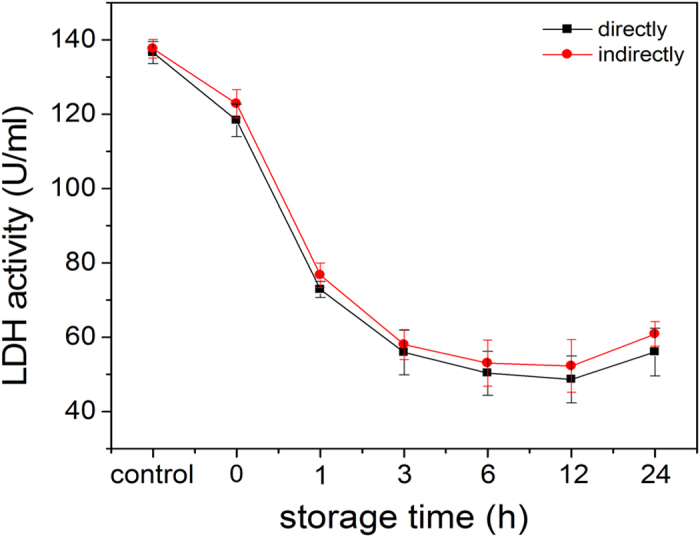
Residual activity of LDH after plasma treatment for 300 s and storage from 0 to 24 h.

To investigate the continuous effects of the LDH activity after plasma treatment, the LDH is treated for 300 s in both the direct and indirect modes. The treated LDH samples are stored at 4 °C and the activity is monitored at different time points. The change in the enzyme activity with storage time is shown in Fig. 7. The enzyme activity declines quickly in the first three hours and then gradually from three to twelve hours. The results indicate that there are continuous effects on the LDH activity regardless of treatment modes attributable to the long-lived RS (hydrogen peroxide, ozone, and nitrate ion) in the PBS created by the DBD plasma. Hydrogen peroxide can oxidize proteins effectively causing the protein to denature as follows^37^:
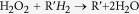


In addition, many amino acids can be modified by ozone^38^, for example,


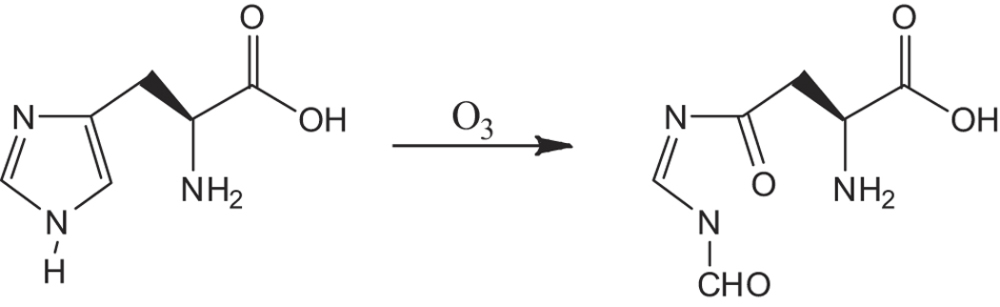


With regard to nitrate ions, conformational change or protein denaturation result from changing the electrical charges^39^,^40^. In comparison with the indirect treatment, the decrease in the LDH activity is smaller in the direct treatment, suggesting that the short-lived RS (OH˙, O_2_^−^, and O_2_ (^1^Δg)) and UV produce only minor effects on the LDH activity. Therefore, the possible mechanism of the continuous effect on protein by DBD plasma is due to hydrogen peroxide^41^,^42^, ozone, and nitrate ions which cause chemical modification of the LDH molecule or between the molecules^25^,^43^,^44^. More experiments are needed to clarify the synergistic effects. As shown in Fig. 7, the LDH enzyme activity exhibits an unexpected increase after storage for 24 hours. This phenomenon indicates some “re-naturing” of the LDH protease after the DBD plasma treatment after a while. A possible reason is that after a long time, the inactivation effect is reversible or the functional domains that are hidden in the interior of the hydrophilic amino acids induced by the DBD plasma are exposed again. This topic will be discussed further later.

### CD analysis

**Figure 8:**
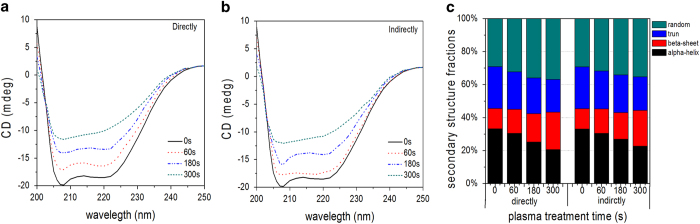
CD spectra and secondary structure percentages of the LDH solution after direct and indirect treatment for 0-300 s: (**a**) LDH after direct treatment, (**b**) LDH after indirect treatment, and (**c**) Exact percentages of the different secondary structures of the LDH protease solution.

Figures 8(a) depict the CD spectra of the LDH after direct and indirect treatment for 0-300 s and one hour after the plasma treatment. The spectra show the typical negative peak at 208 nm corresponding to the percentage of the α-helical structure in the enzyme and a shoulder at 218 nm indicating the percentage of the β-sheet structure. Flattening of these two regions with increasing plasma exposure times indicates reduction in the helical structure and larger β-sheet content. In addition, there is a positive peak at 230 nm and the negative peak represents the turn at 200 nm. The changes illustrate that the secondary structure of the proteins change with treatment time depending on the concentration of RS in the PBS created by the plasma.

The exact percentages of the different structures are calculated from the corrected spectra. The ordered secondary structure of LDH mostly consists of α-helices and turns and the relative content of the β-sheet structure is smaller. As shown in Fig. 8(c), the amounts of turns and α-helix structure decrease drastically with treatment time while the number of random areas and particularly amount of β-sheet structures increase strikingly, indicating that the secondary structure of the LDH enzyme is altered by the plasma. In the LDH solution after the direct treatment, the α-helix content decreases from 33.20 to 20.60%, whereas the β-sheet content increases from 12.30 to 22.70% after treatment for 300 s. In the indirect treatment, the α-helix content decreases from 33.10 to 22.60% and the β-sheet content increases from 12.30 to 21.70%. The loss in the α-helix content and increase in the β-sheet content are similar in both treatment modes, indicating that the long-lived RS in the PBS may play primary roles in the structural changes in the LDH enzyme^35^,^45^,^46^.

In a complex plasma, many reactions can cause conformational and secondary structural changes, but all of them are likely initiated by the inherent RS^36^. These RS are able to cleave peptide bonds and modify amino acid chains thereby producing secondary structure changes. The sulfur-containing amino acids such as cysteine and aromatic amino acids are particularly susceptible. Concerning LDH, the active sites are more flexible compared with the other structure and these amino acids such as Arg-171, His-195, Arg-109 and Asp-168 play a key role in the active sites^47^ and all are involved in the formation of the α-helical structure. Therefore, there is a strong correlation between the enzyme activity and α-helical structure. Oxidation of even one single amino acid in the protein can affect its function. For example, oxidation of histidine-195 at the active side of the LDH can result in loss of activity because the active sites cannot combine with the substrate.

**Figure 9:**
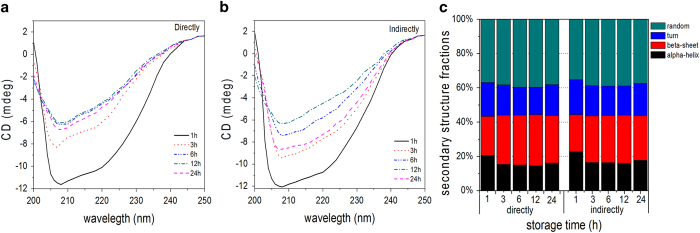
CD spectra and secondary structure percentages of the LDH solution after direct and indirect treatment for 300 s and storage from 0 to 24 h: (**a**) LDH after direct treatment, (**b**) LDH after indirect treatment, and (**c**) Exact percentages of the different secondary structures of the LDH protease solution.

Figures 9(a) show the CD spectra after storing at 4 °C for different time after plasma treatment for 300 s directly and indirectly, respectively. The DBD plasma has continuous effects on the secondary structure of LDH protease but the effects are attenuated after storage and as shown in Fig. 9(c), after storage for 24 h, the second structures exhibit recovery which coincides with that of the enzyme activity further demonstrating that the change in enzyme activity is associated with the change of secondary structure induced by DBD plasma^48^.

### DLS analysis

**Figure 10:**
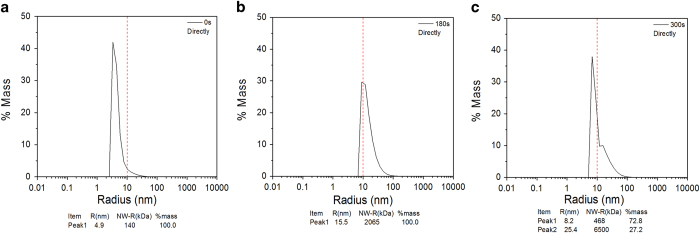
DLS spectra of directly treated LDH for 0-300 s: (**a**) 0 s, (**b**) 180 s, and (**c**) 300 s.

**Figure 11:**
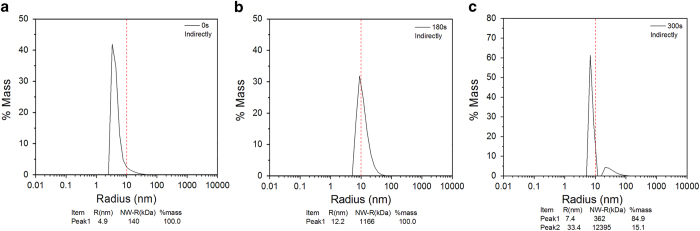
DLS spectra of the indirectly treated LDH for 0-300 s: (**a**) 0 s, (**b**) 180 s, and (**c**) 300 s.

The hydrodynamic radius and particle distribution of the LDH protease solution are determined by means of dynamic light scattering by analyzing the combined behavior to better understand the effects of atmospheric-pressure plasma on LDH. The hydrodynamic radius of the LDH protease solution in the different treatment modes for different treatment time are shown in Figs. 10 and 11, respectively. Figures 10(a) and 11(a) show that the LDH protease solution without the treatment has a mass peak of the contribution ratio of 100%, molecular radius of 4.9 nm, and molecular weight of 140 kDa. As shown in Figs. 10(b) and 11(b), the average hydrodynamic radius increases with treatment time and the particle distribution also changes. The results indicate that the DBD plasma can promote molecular aggregation between the LDH molecules in this concentration range to generate larger and more complex supramolecules^49^,^50^. A possible explanation involves the modification of amino acid, electrostatic interactions, and hydrophobic interactions. Ozone (O_3_)^38^ and particularly hydrogen peroxide (H_2_O_2_)^37^ are able to oxidize the amino acid side chains (cysteine) to form protein–protein cross-linkage^51^,^52^,^53^. In the direct treatment for 180 s, the hydrodynamic radius and molecular weight increase to 15.5 nm and 2056 kDa, respectively (Fig.10 (b)) and these values are larger than those after the indirect treatment (Fig.11 (b)). These differences may be attributed to the effects of UV radiation and short-lived reactive species created by the plasma. Figures 10(c) and 11(c) show two new peaks after plasma treatment for 300 s. A possible mechanism is that weak stability of the irregular supramolecules which further polymerize with increasing treatment time. After a high degree of polymerization, the supramolecules disintegrate to two new polymeric molecules spontaneously^54^,^55^.

**Figure 12:**
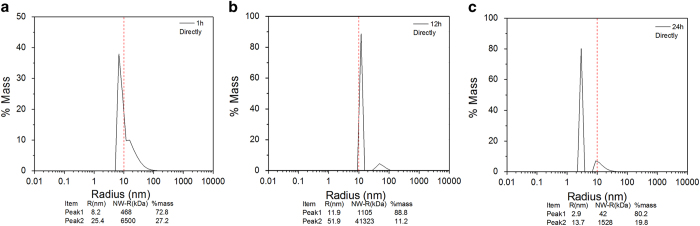
DLS spectra of the directly treated LDH for 300 s and storage for 0, 12, and 24 h: (**a**) 0 h, (**b**) 12 h, and (**c**) 24 h.

**Figure 13:**
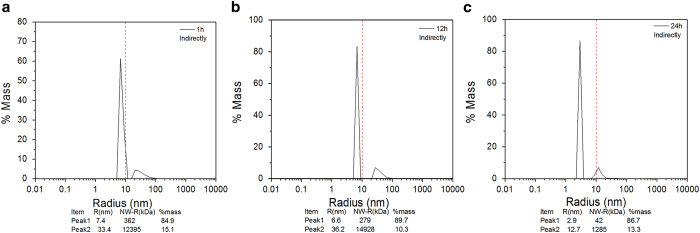
DLS spectra of the indirectly treateld LDH for 300 s and storage for 0, 12, and 24 h: (**a**) 0 h, (**b**) 12 h, and (**c**) 24 h.

The DBD plasma continuous effect on the hydrodynamic radius and particle distribution of LDH protease solution are assessed. As shown in Figs. 10 and 11, polymerization increases in the initial 12 hours and the hydrodynamic radius of the new supramolecules gradually increases (Figs. 12(a); Figs. 13(a)). Moreover, the DLS spectrum acquired after 24 hours shows that there are new small polymers (Fig. 12(c) and 13(c)). A possible explanation is that the supramolecules disintegrate into two new polymeric molecules spontaneously and this is the major reason for the aforementioned enzyme recovery.

**Figure 14:**
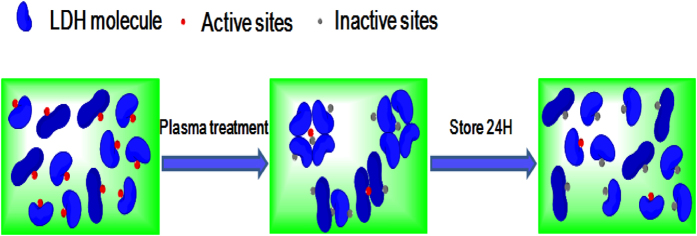
Molecular mechanism showing recovery of the LDH enzyme activity.

Based on the above results, there is a strong correlation between the loss in the enzyme activity and change in the molecular structure depending on the RS produced by DBD plasma. The molecular mechanism of LDH inactivation can be described as follows. First, in the molecule, some important amino acids are modified by the RS and the secondary structure changes, especially the structure of the active center, and the active sites lose recognition and catalytic functions. Secondly, between the molecules, the peptide chains polymerize to form irregular supramolecules which wrap up the small quantity of active sites and hence, the LDH is unable to participate in the catalytic reaction. Aggregation of molecules decreases the enzyme activity and also protects the active sites from being modified by RS. When the supramolecules disintegrate spontaneously after storing for a long time, the protected active sites are exposed again causing recovery as shown in Fig. 14. All in all, the changes in the secondary structure and hydrodynamic radius coincide with the loss and recovery of the enzyme activity. The results suggest that the enzymatic activity change arises from not only intramolecular chemical modification, but also intermolecular aggregation.

### pH and temperature

**Figure 15:**
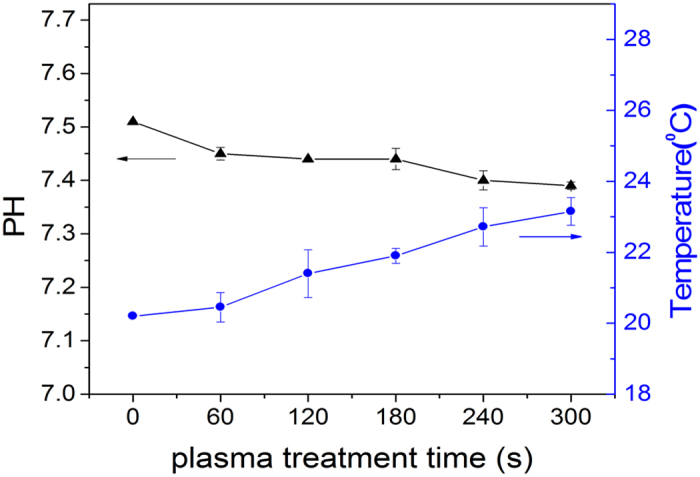
Temperature and pH time profile of the plasma-treated PBS.

The relationship between the temperature and pH of the LDH protease solution with treatment time is shown in Fig. 15. The highest temperature is 24 °C after plasma exposure for 300 s and the pH remains at about 7.5 before and after the plasma treatment because the PBS has the buffering capability. Since the temperature and pH do not reach values that can cause enzyme inactivation, their effects can be neglected.

## Conclusion

A helium-oxygen non-thermal DBD plasma is employed to treat LDH as a model enzyme in PBS. The concentrations of the long-lived RS in the plasma-treated PBS, for instance, hydrogen peroxide, nitrate ions, and ozone, increase with treatment time but decrease with storage time except nitrate. The LDH activity decreases significantly with plasma treatment time or storage time in the first 12 h regardless of treatment modes, but recovers slightly after storing for 24 h. The CD and DLS results suggest the mechanisms to explain the change in the LDH activity. It is likely due to modification of the secondary structure in the molecule and peptide chain polymerization between the molecules as a result of the reactive species created by the DBD plasma. By comparing the direct and indirect plasma treatment, the changes in the LDH activity can be attributed to the RS, especially long-lived ones such as hydrogen peroxide, ozone, and nitrate ion instead of heat, UV radiation, and charged particles.

## Materials and methods

### Discharge Apparatus and Plasma treatment

The atmospheric-pressure DBD plasma is depicted in Fig. 1. The DBD plasma reactors consist of a hollow plexiglass as a reactor chamber on which there are two air inlet and outlet holes. The high-voltage electrode is a 32 mm diameter copper cylinder covered by 1 mm thick quartz glass as the insulating dielectric barrier and the ground electrode is a 37 mm diameter copper cylinder. The discharge gap between the bottom of the quartz glass and sample surface is 5 mm. An alternating current power supply operating in frequencies between 10 and 42 kHz with variable output voltages between 0 and 50 kV (peak to peak) is used.

Helium (99.99% pure) and oxygen (99.99% pure) were the carrier gases and the flow rates regulated by flow meters were 80 L/h and 10 L/h, respectively. In order to eliminate as much air from the reactor chamber as possible, the working gas was bled into the chamber for 5 minutes before the experiment. The non-thermal DBD plasma was generated at voltage of 14 kV (peak to peak) at a frequency of 24 kHz with a discharge power density of about 1 W/cm^2^. One ml of the LDH enzyme solutions in a 35 mm diameter petri dish was treated by the DBD plasma and they were put on ice and stored in a cool bag in order to avoid unintentional inactivation after the treatment. In addition, because the depth of plasma penetration was limited, neither charged particles nor reactive species generated in the plasma could interact directly with the LDH in the solution. Hence, we investigated the LDH treated by direct and indirect plasma treatment to demonstrate the inactivation effects of the reactive species induced by the plasma. In the direct treatment, the plasma was used to treat the LDH enzyme solution whereas in the indirect treatment, the solution without LDH was first exposed to the plasma followed by introduction of the protein to the treated solution instantly.

### Atomic emission spectrometry and molecular-beam mass spectrometry

The optical emission spectra of the helium-oxygen non-thermal dielectric barrier discharge (DBD) plasmas were acquired on the AvaSpec-2048-8-RM spectrometer equipped with gratings of 2,400 grooves/mm at a spectral range between 200 and 900 nm. A molecular-beam mass spectrometer (MBMS, Hiden EQP mass/energy analyzer HPR 60) was operated in the time-averaged mode. The distance between the bottom of the quartz glass of the DBD plasma and orifice of the mass spectrometer was 5 mm.

### Enzymes

LDH (from rabbit muscle, Type V, HM0037, sigma-Aldrich, Shanghai, China) in the mitochondria of eukaryotic cells as tetramer can catalyze dehydrogenation of lactate to pyruvate which can take part in the Krebs cycle to provide energy to the cells or organism. The samples were dissolved in phosphate buffer solution (PBS, pH = 7.5) to an initial concentrations of 5 mg/ml and ultrasonically degassed. The enzyme solutions were divided into small portions of 1 ml, put into 1.5 ml centrifuge tubes, and stored at −20 °C until usage. These stock solutions were prepared once for the whole study.

### Enzyme activity assays

The LDH activity was monitored using the double antibody sandwich method. The purified rabbit LDH antibody was used to coat the microtiter plate wells and make the solid-phase antibody. The LDH antibody was mixed with horseradish peroxidase (HRP) to form the antibody-antigen-enzyme-antibody complex. After washing and addition of tetramethylbenzidine (TMB), the solution turned blue due to the HRP enzyme-catalyzed reaction. Sulfuric acid was added and the color change was monitored spectrophotometrically at 450 nm. The concentration of the rabbit LDH was determined by comparing the optical density (OD) with the standard curve. The absorbance was determined with respect to a value on a microplate reader (Varioskan Flash, Thermo Scientific, Finland). The measurement was completed within 15 min after adding the solution and the LDH liquid samples were diluted five times prior to the measurement. The enzyme activity was expressed as the absolute one and the error represented the standard deviation derived from at least six independent measurements.

### Circular dichroism spectroscopy

The CD spectra were acquired in the UV range from 200 to 250 nm on a Jasco-810-CD spectropolarimeter (Japan Spectroscopic Company, Tokyo, Japan) with a 500 μl sample quartz cup. The LDH enzyme solutions were diluted from 5 mg/ml to 0.15 mg/ml before the CD spectra were obtained. The quartz sample cup was cleaned by doubly distilled water and the secondary structure fractions were calculated from the spectra using the CD analysis software. The absorbance was also measured at 25 °C.

### Dynamic light scattering (DLS)

The samples were injected into a sample cell (50 μl) in the Dynapro-MS800 (ATC, England) instrument illuminated by a 25 mW and 750 nm solid-state laser at a fixed scattering angle θ = 90^o^ equipped with a digital autocorrelator. The delay time was linearly spaced to sample the broad distributions properly. The measurements were performed at 25 °C and the data were then collected after temperature stabilization for 10 to 15 min. Before the test, the samples were filtered using inorganic membrane filters (whatman, Anatop10 Plus, 0.22 μm), centrifuged at 10,000 rpm for 10 minutes, and degassed while attention was paid to make sure there were no bubbles in the samples.

The particle size was related to the translational diffusivity (D) and the instrument calculated the diffusivity (D) of the molecules in the sample based on autocorrelation of the scattered light intensity. The hydrodynamic radius of the molecules was derived from D using a uniform sphere and Stokes–Einstein equation, D = kT/6 πηr, where k is Boltzmann’s constant, T is the absolute temperature, η is the viscosity of the liquid in which the particle is moving, and r is the hydrodynamic diameter^56^. This equation assumes that the particles move independently of each another. Finally, the DynaPro-MS800 estimated the molecular weight according to the existing model of molecular radius / molecular weight and the distribution of particles in the solution.

### Measurement of RS in PBS induced by plasma

When the plasma reacts with the liquid such as deionized water or PBS, RS are produced but direct monitoring of RS in the liquid is difficult due to the short half-life and high reactivity. Here, we only measured the concentration of the long-lived RS such as hydrogen peroxide, nitrate, and ozone in the plasma-treated liquid (PBS solution) spectrophotometrically on the PhotoLab 6100(WTW, Germany). The test kits were 18789, 09713, and 00607 respectively and the test methods were according to the WTW company website^57^.

### pH and temperature

For the LDH protease solutions, the temperature was measured by an infrared thermometer (Raytek ST20 XB) and regular thermometer. The pH values were determined using a Basic PH meter (PB-10, Sartorius, Germany). The temperature and pH of the enzyme solutions were measured simultaneously.

## Author Contributions

W.X. and C.C. led the project and supervised all experiments. H.Z., Z.X., J.S., X.L., L.D. J.M., Y.L., Q.S. and Z.Z conducted experiments and measurements. W.X., C.C. and P.C. co-led data analysis and physical interpretations. All authors discussed the results. H.Z, W.X., C.C. and P.C. co-wrote the manuscript.

## Additional Information

**How to cite this article**: Zhang, H. *et al.* Effects and Mechanism of Atmospheric-Pressure Dielectric Barrier Discharge Cold Plasma on Lactate Dehydrogenase (LDH) Enzyme. *Sci. Rep.* **5,** 10031; doi: 10.1038/srep10031 (2015).

## Figures and Tables

**Table 1:** DBD plasma production of biologically relevant RS.

**RS**	**Formula**	**Plasma reactions**
Hydrogen peroxide	H_2_O_2_	H_2_O^+^ + H_2_O → OH• + H_3_O^+^
		OH• + OH• → H_2_O_2_
Ozone	O_3_	O + O + M → O_2_ + M^a^)
		O + O_2_ + O_2_ → O_3_ + O_2_
		O + O_2_ → O_2_+O_2_
Nitrate	NO_3_^−^	NO + O_3_ → NO_2_ + O_2_
		2NO_2_ + H_2_O→ HNO_2_ + HNO_3_

M^a^), Third body particle.
